# An Interprofessional Quality Improvement Project to Implement Maternal/Infant Skin-to-Skin Contact During Cesarean Delivery

**DOI:** 10.1111/1552-6909.12469

**Published:** 2014-06-30

**Authors:** Karen Brady, Denise Bulpitt, Caren Chiarelli

**Affiliations:** Center at St. Vincent's Medical CenterBridgeport, CT; University of Texas Southwestern Medical CenterDallas, TX

**Keywords:** Baby-Friendly, interprofessional, skin-to-skin contact, cesarean delivery, perinatal core measures, exclusive breastfeeding

## Abstract

Immediate skin-to-skin contact between a mother and her newborn has been associated with successful breastfeeding outcomes. One of the challenges nurses face in promoting skin-to-skin occurs in the operating room during a cesarean delivery. Utilizing an interprofessional approach for this quality improvement project, we successfully implemented skin-to-skin contact for all eligible mother/infant couplets after cesarean birth. Exclusive breastfeeding rates for these women increased as a result.

The benefits of breastfeeding are recognized throughout the world. Breastfeeding benefits not only the mother and her infant, but also society as a whole when taking into account the decrease in illness and associated costs (Bartick & Reinhold, 2010). The World Health Organization (WHO; 2013) and Baby-Friendly USA (2011) encourage all hospitals and birthing centers to institute the Ten Steps to Successful Breastfeeding (Table 1) as a means to promote and support breastfeeding for all mothers. As of April 2013, 154 hospitals and birthing centers in the United States had achieved the Baby-Friendly designation (Baby Friendly USA, 2014), identifying them as centers that support and promote breastfeeding as the gold standard for infant nutrition.

**Table 1:** Baby-Friendly USA Ten Steps to Successful Breastfeeding

Step Number	Success Indicator
1.	Have a written breastfeeding policy that is routinely communicated to all health care staff.
2.	Train all health care staff in the skills necessary to implement this policy.
3.	Inform all pregnant women about the benefits and management of breastfeeding.
4.	Help mothers initiate breastfeeding with one hour of birth.
5.	Show mothers how to breastfeed and how to maintain lactation, even if they are separated from their infants.
6.	Give infants no food or drink other than breast milk, unless medically indicated.
7.	Practice rooming in - allow mothers and infants to remain together 24 hours a day.
8.	Encourage breastfeeding on demand.
9.	Give no pacifiers or artificial nipples to breastfeeding infants.
10.	Foster the establishment of breastfeeding support groups and refer mothers to them on discharge from the hospital or birth center.

*Note.* From Baby-Friendly USA, Inc. Used with permission from United Nations International Children's Emergency Fund (UNICEF).

Baby-Friendly USA provides a 10-step model of practice that supports specific opportunities for continuous improvement. Hospitals designated as Baby-Friendly regularly evaluate evidence-based practice (EBP) regarding breastfeeding to provide women and their infants with the best opportunities for breastfeeding success. Step Four of the Baby-Friendly Ten Steps addresses the importance of skin-to-skin contact (SSC) immediately after birth, with the goal of initiation of breastfeeding within an hour of birth (Baby-Friendly USA, 2011). For hospitals attempting to receive the Baby-Friendly designation, this particular step can present many challenges, as it involves a change in the model of care and routine nursing practices.

Although every member of the patient care team can receive education and speak to the benefits of early SSC for mother and infant, implementing this practice takes a coordinated effort. There are numerous barriers to overcome, particularly for women undergoing cesarean births. With the cesarean birth rate at in the United States approximating 33% (Hamilton, Martin, & Ventura, 2012), it is imperative that all women and their infants be provided with the best opportunity for breastfeeding success. This best opportunity for success includes early initiation of SSC after birth. The purpose of this article is to outline the interprofessional process necessary to implement SSC in the operating room (OR) as a quality improvement project.

Breastfeeding exclusivity rates may be lower among women who experience cesarean delivery. Implementing skin-to-skin contact in the operating room can affect exclusive breastfeeding.

## Setting

This quality improvement project was conducted at a 473-bed community teaching and referral hospital located in Bridgeport, Connecticut. The Family Birthing Center (FBC), consisting of labor and delivery, postpartum couplet care, and a Level IIb nursery, provides care for approximately 1,200 women and their infants each year. There are currently 57 RNs, 8 clinical care associates/certified surgical technicians, 2 lactation consultants, 1 clinical nurse leader, 1 clinical nurse educator, and a nurse manager working in the FBC. The entire FBC staff, including physicians, participated in the process to achieve the Baby-Friendly designation. As part of the initial training, all nursing staff was required to attend a 2-day workshop titled “Ten Steps to Successful Breastfeeding – Support Specialist Program” and to spend supervised hours with a lactation consultant (Baby-Friendly USA, 2011). Education for physicians included grand rounds presentations, an online learning module titled Breastfeeding Exercises, and some individual sessions with a lactation consultant.

## Project Background

The Joint Commission (TJC) defines *exclusive breastfeeding* as “a newborn receiving only breast milk and no other liquids or solids except for drops or syrups consisting of vitamins, minerals, or medicines” (The Joint Commission [TJC], 2013, p. 67). Exclusive breastfeeding while in the hospital has been associated with an increase in the overall duration of breastfeeding (Murray, Ricketts, & Dellaport, 2007). Increased duration of breastfeeding is one of the objectives of Healthy People 2020, which are defined by the U.S. Department of Health and Human Services (2010, November). Our FBC data indicated that for infants born via cesarean in the year 2011, 9% were discharged home exclusively breastfeeding.

The implementation of Step Four, which is to help mothers initiate breastfeeding within one hour of birth (Baby-Friendly USA, 2011), presented an operational challenge that the staff was able to overcome with vaginal births but not cesareans. The initial challenge with this step involved the practice of initiating SSC immediately after birth. According to the guidelines of Step Four, infants should be placed in continuous SSC with their mothers from immediately after birth for at least one hour until completion of the first breastfeed. For infants born via cesarean, the additional qualifier in Step Four is that the infant is placed with the mother once the mother is responsive and alert (Baby-Friendly USA, 2011). Routine nursing practices and newborn admission assessments were reasons cited for the initial resistance to SSC by members of the nursing staff. Through staff education, peer coaching, and role modeling, SSC became the standard of care for vaginal births.

Upon acceptance of SSC for vaginal births, the next step was to incorporate this model of care into cesarean deliveries. At the time of the Baby-Friendly designation, the practice of SSC for women and their infants experiencing cesarean deliveries occurred in the postanesthesia care unit. Upon acceptance of the practice change with SSC in the postanesthesia care unit, placing the infant in SSC with the mother in the operating room (OR) during a cesarean delivery was the final step to full implementation of Step Four. Some members of the medical and nursing staffs expressed concerns about the safety of placing an infant SSC in the OR. Specific concerns included temperature stability, need for increased surveillance of the mother and infant during a cesarean delivery, and the ability to follow recommended staffing guidelines to provide a nurse to care for the infant while facilitating SSC in the OR.

An important piece of the implementation plan for SSC during a cesarean delivery was the utilization of a transition nurse. The transition nurse role was established during the process to become Baby-Friendly. Based on the recommended staffing guidelines of one nurse to one patient for circulating during a cesarean delivery, an additional nurse was needed to care for the newborn (American Academy of Pediatrics & American College of Obstetricians, 2012). Additionally, the Association of Women's Health, Obstetric and Neonatal Nurses (AWHONN; 2010) staffing guidelines recommend a nurse to patient ratio of two to one at birth, one nurse for the mother and one nurse for the newborn.

The role and responsibilities of the transition nurse were discussed and defined with input from nurses and physicians, as this change in the model of care affected the entire team. The transition nurse's primary responsibility is to attend all births and provide care for the newborn infant at the bedside, rather than removing the newborn to the nursery. The transition nurse assists new mothers with immediate SSC. The initial assessment and routine tasks are delayed until completion of the first breastfeed.

There are two key committees that were integral to the successful achievement of SSC in the OR. Being interprofessional in nature, these committees were able to monitor progress and obstacles in processes and acceptance of change by team members. These two committees, with distinct purposes, are the Perinatal Safety and Patient Satisfaction (PSP) committee and the Baby-Friendly Committee. Each of these committees played an active role in the implementation of SSC in the OR and is detailed in Table 2.

**Table 2:** Committee Characteristics Related to SSC Initiative

Name of Committee	Membership	Oversight	Reporting Structure
Baby-Friendly Committee	Interdisciplinary members of the Perinatal Team, Quality Improvement Specialist	Baby-Friendly Metrics, Yearly Action Plan, Review of Policies and Procedures of Baby-Friendly.	Summary shared at staff meetings, Monthly Perinatal Safety and Patient Satisfaction Committee, pediatric and obstetrics department meetings.
Perinatal Safety and Patient Satisfaction Committee	Interdisciplinary members of the Perinatal Team, Security, Risk Management, Quality Improvement Specialist.	Maternal Child Health Scorecard metrics, Joint Commission Perinatal Core Measures, Delivery Data, Immunization data, patient satisfaction scores, bundle compliance and crisis simulation drills.	Hospital Quality Council, Monthly meeting summaries shared at Family Birthing Center staff meetings and pediatric and obstetrics department meetings.

The Perinatal Safety and Patient Satisfaction (PSP) committee originated as part of an Institute for Healthcare Improvement (IHI) safety initiative, in early 2006, and is recognized as a change agent for practice improvements in the FBC. Responsibilities of this committee include bundle compliance, crisis simulation drills, patient satisfaction monitoring, and oversight of all aspects of patient safety on the unit. An additional function of this committee is to review the perinatal score card and compliance with TJC's (2013) Perinatal Core Measures. Exclusive breastfeeding is one of the core measures, therefore collaborating with this committee was essential to the success of the quality improvement project. Committee membership includes nurses, obstetricians, neonatologists, pediatricians, anesthesia providers, quality improvement and risk management staff, and security. This team approach provided the opportunity to evaluate SSC in the OR, with each discipline offering a unique perspective on patient safety as well as practical solutions to overcoming perceived barriers.

The Baby-Friendly Committee was created in the fall of 2006, with the goal of bringing the Baby-Friendly designation to our institution. Committee members include the medical directors of obstetrics and pediatrics, the nurse manager, clinical nurse leader, clinical nurse educator, lactation consultants, staff nurses, and a hospital quality improvement specialist. Upon being designated Baby-Friendly, the committee continued to meet monthly to ensure compliance with the Ten Steps and work on maintenance of the designation. Additionally, the committee reviews Baby-Friendly statistics, including TJC (2013) Exclusive Breast Milk Feeding PC-05. Statistics on SSC and infants breastfed in the first hour of life are also evaluated monthly, to monitor trends and discuss opportunities to improve clinical practice and breastfeeding outcomes.

## Method

The plan, do, study, act (PDSA) method was used to implement small tests of change in a disciplined fashion and to assess the feasibility of initiating SSC contact in the OR (Nelson, Batalden, & Godfrey, 2007). The Transforming Care at the Bedside workgroup from IHI suggests that when planning a test of change three conditions should exist: staff should be willing to try it, they should believe it's a good idea, and there should be a low cost of failure should the test fail (Rutherford et al., 2008). The PDSA method provided ongoing tests of change and revisions to the process on a small scale, while incorporating the interprofessional collaboration needed for the overall success of the project.

## Plan

The Baby-Friendly Committee analyzed current practices on the unit to identify opportunities that could positively affect breastfeeding exclusivity rates at discharge. The initial step was to build support of the initiative through evidence. Members undertook literature searches and information was dispersed among the staff about evidence that was found. In particular, team members researched and discussed articles regarding increasing trends in cesarean section rates, lower exclusive breastfeeding rates among women who had a cesarean delivery, and SSC in the OR. Publications of special interest were brought to a formal journal club for discussion. One article in particular, by Hung and Berg in 2011, was reviewed as it discussed one California hospital's quality improvement project to implement SSC in the OR from a nursing perspective. Collectively the time spent on exploring the evidence and discussing it in an interprofessional format supported the “ownership” of team members and established excitement about the project.

As plans were formulated to begin this performance improvement project, it became evident that implementing SSC in the OR would require an interprofessional team approach. Key members of the interprofessional team included nurses and scrub technicians, obstetricians, anesthesiologists and certified registered nurse anesthetists, neonatologists and pediatricians, lactation consultants, and last, patients themselves. Each of these disciplines brought a unique perspective and set of challenges to the practice change. A few of obstetricians were concerned that aseptic technique in the OR would be jeopardized if the infant's feet should slip beneath the drapes and into the surgical field. Various anesthesia personnel thought they might be responsible for monitoring an infant while in SSC with its mother. Many nurses worried the infant might get cold stressed in the cool OR environment, or that the mother wouldn't be able to sufficiently hold her infant in SSC in the OR. Last, a few pediatricians shared their thoughts on the newborn transition period and the nurse's ability to monitor the infant while in SSC with the mother. These concerns were discussed with each discipline at individual department meetings and collectively at the PSP and Baby-Friendly Committee meetings.

A solid educational plan was another component of the implementation design. Education across all disciplines is necessary for facilitating change in any setting. Baby-Friendly USA (2011) details a prescribed 15 hour curriculum for nursing staff working with new mothers. Currently, education of nursing staff regarding SSC in the OR begins in orientation with new staff. As part of breastfeeding orientation, education is provided on what SSC means, the benefits of SSC, and how to facilitate SSC for vaginal births and cesarean deliveries. Placing infants in SSC regardless of feeding choice or delivery method is a cornerstone of this education curriculum (Baby-Friendly USA, 2011). Breastfeeding and SSC immediately after birth are the standard of care. Newly hired physicians complete an online learning module on breastfeeding, which includes information on Baby-Friendly USA and the Ten Steps. Lactation updates are a standing agenda item for the monthly FBC staff meeting, as well as the monthly meetings of the department of obstetrics and pediatrics, to provide ongoing education as needed. Nursing education also includes dissemination of the practice change and the support of EBP through the Baby-Friendly Committee, Unit Practice Council, and journal club.

Patient education plays a vital role in partnering with patients to implement an individualized plan of care. Mothers and their significant others can be seen as a barrier to this model of care. Prenatal education should include the practice of SSC, to avoid risking a delay in the initiation of SSC after birth (Rowe-Murray & Fisher, 2002). This education will allow the mother to understand the benefits of SSC after birth regardless of her feeding preference. Although the majority of women are able to participate in SSC in the OR, a mother must also understand the possibility of her inability to hold her infant due to instability on her part, or on the part of her infant. Instances of general anesthesia, intraoperative pain or nausea and vomiting, or infants in need of resuscitation or a higher level of care will result in delaying SSC for the mother/infant couplet. Finally, a mother might feel modest about exposing her breasts, due to cultural or personal reasons, and this barrier needs to be approached with sensitivity and care.

Prenatal education on SSC is offered in a variety of ways. At this institution, it has been incorporated into childbirth education class, prenatal breastfeeding class, baby care class, sibling class, and prenatal tours. The process of placing the infant on the mother in SSC as soon as possible after birth is not only described, but also demonstrated in patient education videos and printed information. It is promoted as the standard of practice. Benefits, including health benefits for mother and infant, as well as patient satisfaction are reviewed. Additionally, SSC can be incorporated into prenatal visits, especially if a surgical delivery is anticipated. Discussing SSC in the OR as a means of normalizing the birth experience can have a positive effect on a patient's anxiety level. The local Women, Infants and Children (WIC) office have breastfeeding coordinators discuss SSC with their clients during prenatal visits. Referrals are made to the hospital lactation consultants for follow-up if further education is needed.

The final piece of the patient education process takes place upon admission to the FBC. At this time, prenatal education of SSC is reinforced with patients who have attended classes, or introduced to patients who are new to the concept. This education is provided by the nursing staff and is documented in the electronic medical record (EMR). A key element in the planning was enhancements made to the EMR to allow for ease of documentation and data collection. When admission education is complete, nurses document the education provided, as well as the patient's agreement to SSC, in the EMR.

## Do

With the ground work laid for implementing SSC in the OR, it was time to begin a test of change. Two nurse champions volunteered to pilot SSC in the OR. For the first test of change, the transition nurse spoke with members of the OR team prior to the start of the case to discuss the possibility of initiating SSC in the OR. With the team in agreement, the nurse then spoke with the patient preoperatively, provided education on SSC, and obtained verbal consent from the patient to proceed. Following birth, the infant was placed under the radiant warmer for a brief period of observation by the pediatrician, as per the usual OR routine at that time. After 5 minutes of observation, the transition nurse prepared the mother for SSC and placed the infant on her chest, where the infant remained for the duration of the operation. Following AWHONN staffing guidelines (2010), the transition nurse remained with the infant the entire time, while the circulating nurse attended to the needs of the surgical team and the mother. The following day, the second nurse champion, using the same procedure, initiated SSC in the OR.

Nurse champions, collaborating interprofessionally with the operating room team, were successful in implementing skin-to-skin contact in the operating room.

An informal discussion followed each of these two tests of change. The patients and the transition nurses reported a high degree of satisfaction with this change in practice. Positive comments included patient satisfaction at being able to hold her infant earlier, increased breastfeeding within the first hour, and surprise from the nurses at the ease of placing a newborn SSC in the OR. Based on the success of the first two tests of change and the feedback received from the team, the plan was to continue with a core group of engaged team members to further identify potential barriers and build on successes. Multiple tests of change occurred until implementation of the process was complete.

As part of the learning process, staff was encouraged to talk about their experiences with initiating SSC, and practice SSC in the OR with every cesarean delivery. Nurse champions, who have been shown to successfully affect workplace practices, acted as peer coaches and engaged additional staff members in the initiation of SSC in the OR (White, 2011). With the help of these champions, acceptance of SSC in the OR increased. Members of the team were empowered to implement any changes to the process that were easy to do and resulted in improvements.

Simultaneously with the tests of change, preparations were under way to disseminate information on the practice of SSC in the OR to the other members of the interprofessional team. A list of hospital committees was compiled that included members of the interprofessional team needed for success. The list included departmental meetings for the obstetricians, pediatricians, anesthesia personnel, FBC staff meetings, Unit Practice Council meetings, and the PSP committee. Each of the key team members provided their own insight into the process and their feedback was instrumental in overcoming the barriers identified. Evidence in the literature was used to support the practice change and reassure members of the OR team that their concerns were being acknowledged and addressed. At one department meeting, various positions for SSC were demonstrated to team members, to assure them that aseptic technique could be maintained throughout the surgery.

Thermoregulation was a concern for many members of the team, but a study by Gouchon and colleagues (2010) provided information to overcome this barrier. The results of their study showed that infants born via cesarean section who received SSC were not at risk for developing hypothermia compared to infants who did not receive SSC. Additionally, current neonatal resuscitation guidelines allow for SSC to provide thermoregulation for infants who are term, breathing or crying, and exhibit good muscle tone (American Academy of Pediatrics and American Heart Association, 2011).

Providing a safe environment through adequate resources was vital to the success of the project. The role of the transition nurse, as the primary care provider for the infant based on staffing guidelines (American Academy of Pediatrics and American College of Obstetricians, 2012; AWHONN, 2010), was reinforced with all disciplines and key to the acceptance of the practice. The lactation consultant and the clinical nurse leader attended a department of anesthesia meeting to discuss the concept of SSC in the OR and the anesthesia provider's role in the process. Once concerned members of the group had a clear understanding of the role the transition nurse and her sole responsibility of monitoring the infant, and that the function of the circulating nurse would remain unchanged, they were more accepting of the practice change. The pediatricians were also reassured, knowing that the infant would receive constant surveillance while in SSC with its mother.

## Study

Evaluation of the project was the shared responsibility of established committees and project specific designees. Initial monitoring of the change in practice took place via chart review and feedback between select members of the Baby-Friendly Committee and members of the OR team. It was through the work of the PSP and Baby-Friendly Committee that many of the details of the process were finalized utilizing EBP. Both committees continued to meet monthly and tracked the data that were being collected on initiation of SSC in the OR and breastfeeding exclusivity rates. These monthly meetings provided the opportunity for all disciplines involved to share feedback and troubleshoot any problems encountered, such as equipment that created barriers and staff bias in regards to the change in practice.

It was also a time to share positive case reports and acknowledge the efforts of the team. As more nurses began to incorporate this practice into the OR routine, feedback from the team was used to make improvements to the process. The logistics of positioning the baby with the mother to ensure sterility during the case, the role of the transition nurse continually at the family's side for safety, placing the electrocardiogram leads off to the side to accommodate the infant SSC on the mother, and the timing of the initiation of SSC are examples of some of the innovations based on input from the team. As time went on, the feedback became increasingly positive as acceptance of the process change became part of the normal OR routine.

## Act

After 6 months of preparation, the FBC staff fully implemented SSC for patients undergoing a cesarean delivery. All members of the team cooperated with the practice change. Policies, and procedures had been revised, enhancements were made to the EMR, and patient safety needs addressed. SSC in the operating room was to be initiated for all medically stable mothers and infants.

## Methods of Evaluation

A key aspect of evaluation is data analysis. Initially, anecdotal data identified barriers previously unknown to the committees, as well as spread success stories. Once a few tests of change had been completed, it became important to obtain more concrete data. The Baby-Friendly Committee selected the items that were needed for data collection and changes were made to the EMR to capture these data. An item was created to capture instances of SSC in the OR, as well as the time of initiation of SSC. For ease of documentation by the transition nurse, each of these items was added to the newborn assessment screen on the delivery summary. With the completion of these changes, SSC in the OR was added to the lactation statistics report, making the data available for monthly review.

A second key component of the data analysis was to create a report to monitor trends. Through the use of the EMR, a breastfeeding statistics report was generated that tracked the patient-specific details of instances of SSC in the OR. The lactation consultants, along with the clinical nurse educator, tracked the data from this report and audited charts to monitor the progress of this quality improvement project. This detailed report contained specific information extracted from each delivery including method of delivery, delivery date/time, anesthesia type, gestational age, feeding preference, SSC in OR, breastfed in first hour, delivery nurse, as well as the disposition of the infant (i.e., mother/baby care, nursery for observation or special care nursery). This allowed the team to drill down for more information than the lactation statistics report offered, and provided the opportunity to identify trends should a decline in SSC in the OR occur.

In addition to tracking individual patient data on initiating SSC in the OR, the report identified patients that did not initiate SSC. Each individual case was analyzed to determine why SSC was not initiated. Reasons for noninitiation of SSC included: patient refusal, condition of mother and/or infant, bottle feeding, and resistance among team members. Staff members who initiated SSC for their patients were praised for their efforts and had their success stories shared with the staff. Discussions were held with team members who did not initiate SSC to determine any barriers they encountered, and provided on-the-spot trouble shooting or reeducation as needed.

The final piece of the data collection was anecdotal experiences shared by the patients with the lactation consultants during their postpartum rounding. Patients have remarked on how SSC in the OR normalized their birth experience. In addition, several mothers indicated that they really enjoyed having the baby with them SSC in the OR, and it was much better than when they had their last baby. When patients praised the efforts of the staff in the OR for placing the babies SSC, this feedback was shared with the staff.

Data analyses and educational support among disciplines were key factors in the successful implementation of skin-to-skin contact in the OR.

## Outcomes

The major goals of this performance improvement project were to implement SSC in the OR and increase exclusive breastfeeding rates at discharge. A small test of change began with two women and their infants participating in SSC in the OR. At the completion of the first month of full implementation, 43% of women undergoing a cesarean delivery experienced SSC in the OR. The FBC goal for 2013 is 80% and will be reevaluated upon achievement and sustainment of that goal.

**Figure 1:**
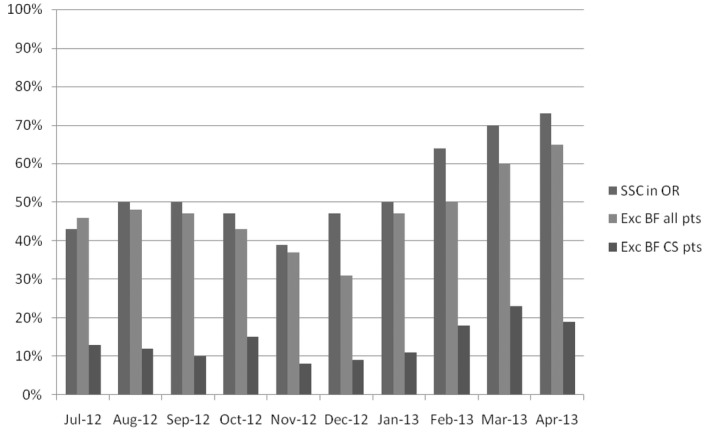
Skin-to-skin in the operating room compared with exclusive breastfeeding rates July 2012 –- April 2013. SSC = skin to skin contact; OR = operating room; Exc BF all pts = exclusive breastfeeding for all patients; Exc BF CS = exclusive breastfeeding for patients with cesarean birth.

Figure 1 represents the first 10 months of data for SSC in the OR, exclusive breastfeeding rates, and exclusive breastfeeding rates among patients experiencing a cesarean delivery. As cases of SSC in the OR increased, there was an increase in exclusive breastfeeding rates as well, from 30% in December 2012 to 63% in April 2013. Exclusive breastfeeding rates for women undergoing a cesarean delivery increased from 8% in December 2012 to 19% in April 2013.

As with all new process changes, continued surveillance is required to ensure that the change has been fully embraced and incorporated into practice. The Baby-Friendly Committee and the PSP Committee will continue to monitor the data to identify trends and provide reeducation as needed. Further monitoring through team observation and chart audits will be utilized to sustain the practice change. Through collaboration of the interprofessional team, this project was successfully implemented. It will require their continued efforts to maintain its success.

## Conclusion

Hospitals across the country have been challenged to improve breastfeeding initiation rates and sustained breastfeeding at discharge. It has become a national health care priority. To meet these challenges, more and more hospitals are attempting to achieve the Baby-Friendly designation. Implementing Step Four of the Ten Steps, particularly in the OR, requires a well-coordinated interprofessional collaborative effort to achieve success. The barriers to the implementation of Step Four in the OR were overcome with the support of the interprofessional team. Continuous monitoring of staff compliance and data collection will ensure sustainability of the practice change. Through communication and collaboration, the healthcare team in the FBC was able to overcome the barriers of SSC in the OR and have succeeded in fully implementing Step Four of the Baby-Friendly Ten Steps.
